# Strengthening nurturing care by supporting caregiver engagement in early childhood stimulation activities

**DOI:** 10.1016/j.jped.2026.101584

**Published:** 2026-07-15

**Authors:** Gabriela Buccini

**Affiliations:** University of Nevada, School of Public Health, Department of Social & Behavioral Health, Las Vegas, USA

Enabling nurturing care for early childhood development requires stimulating and safe environments that are sensitive to children’s health and nutrition needs, and that provide them with timely and developmentally appropriate learning experiences, together with responsive and emotionally stimulating interactions [1,2]. Recognizing the central role of these conditions in promoting healthy early childhood development, the World Health Organization (WHO), UNICEF, and the World Bank launched the Nurturing Care Framework, which identifies five interrelated components – good health, adequate nutrition, safety and security, responsive caregiving, and opportunities for early learning – as essential conditions for every child to survive and thrive [1,2].

Within the Nurturing Care Framework, caregiver engagement in developmentally appropriate stimulation activities such as reading, storytelling, singing, playing, and other forms of responsive interaction represents a fundamental pathway through which nurturing environments influence early childhood development [3]. Regular caregiver engagement in stimulation activities, whether structured or unstructured, fosters children’s cognitive, emotional, and behavioral capacities that underpin lifelong learning while protecting children from the adverse effects of prolonged stress [4,5]. Growing evidence from developmental neuroscience further demonstrates that play promotes language acquisition, strengthens executive functioning, facilitates adaptive responses to stress, and contributes to healthy brain development [[5], [6], [7]]. Beyond its benefits for children, caregiver engagement in stimulation activities has also been shown to reduce stress and promote emotional well-being among adults, reinforcing its importance as a central component of nurturing family environments [8].

In this context, Teixeira et al. [9] sought to identify the determinants of caregiver engagement in child stimulation activities within the family environment. The authors hypothesized that families experiencing more favorable socioeconomic conditions, together with greater access to health care, adequate nutrition, safety, responsive caregiving, and opportunities for early learning, would be more likely to engage in stimulation activities than families experiencing greater socioeconomic disadvantage [9]. To test this hypothesis, the study analyzed data from 365 mother–child dyads recruited from six Primary Health Care units in a mid-sized municipality in the state of São Paulo, Brazil. These data originated from a quasi-experimental pilot study designed to strengthen developmental surveillance through training primary healthcare professionals in the use of Brazil's Child Health Handbook, with caregiver interviews conducted before and after implementation of the training [9]. Caregiver engagement in stimulation activities was assessed based on participation in four or more stimulation activities, including reading, storytelling, singing, walking, playing, and naming, counting, or drawing with the child during the three days preceding the interview [9]. To examine the determinants of these behaviors, the authors applied an adapted multigenerational life-course framework nested within the Nurturing Care Framework, allowing distal, intermediate, and proximal determinants to compete simultaneously for explanatory power within the same analytical model.

The findings challenge conventional assumptions regarding the determinants of caregiver engagement in stimulation activities. Rather than broader socioeconomic determinants, the strongest predictors of caregiver engagement in four or more stimulation activities were three proximal factors within the NCF domain of opportunities for early learning: the availability of books in the home (IRR 1.53, 95% CI 1.23–1.92), child daycare attendance (IRR 1.42, 95% CI 1.15–1.75), and opportunities to play with toys or household objects (IRR 1.24, 95% CI 1.02–1.51) [9]. Collectively, these findings suggest that access to tangible developmental resources may exert greater influence on caregiver engagement in stimulation activities than the broader socioeconomic determinants traditionally considered the primary drivers of nurturing care. From a public health perspective, this represents an important actionable finding, highlighting modifiable factors that can be strengthened through primary health care to promote early childhood development. While socioeconomic disadvantage remains a well-established determinant of early childhood development [1,10,11], the resources identified in this study, including books, toys, opportunities for play, and access to early childhood education, represent tangible and modifiable factors that can be promoted through primary health care. This interpretation echoes evidence from literacy-promotion initiatives such as *Reach Out and Read* in the United States, which has consistently demonstrated that providing caregivers with books during routine pediatric visits can positively influence home literacy practices, particularly shared reading [12].

Beyond the availability of developmental resources, the study also highlights the access to healthcare system as a modifiable context for promoting nurturing care. Receiving information about child development during primary healthcare and pediatrician visits was associated with a 53% higher prevalence of reading with children (IRR 1.53, 95% CI 1.04; 2.26), although this association did not extend to the composite outcome of engagement in four or more stimulation activities. Likewise, maternal concern regarding child development was associated with more frequent play (IRR 1.10, 95% CI 1.00; 1.21). When considered alongside the independent association between daycare attendance and several stimulation activities, including reading, naming, counting, drawing, and play [9], these findings suggest two complementary pathways for promoting caregiver engagement: (a) the guidance families receive from healthcare professionals and (b) the developmental opportunities children experience within early childhood education settings. Both pathways represent opportunities for intervention that are readily accessible within existing Brazilian health, social, and education systems [13]. Unlike distal socioeconomic determinants that often require sustained policy and economic reforms, strengthening developmental counseling during routine consultations and facilitating access to community resources are interventions that pediatricians [14], and primary healthcare teams can implement within current models of care.

The study's limitations appropriately temper the interpretation of its findings. The cross-sectional design precludes causal inference, and several key associations are likely bidirectional, reflecting both determinants and consequences of caregiver engagement. In addition, the convenience sample from a relatively well-resourced municipality, reliance on maternal self-report, and inability to account for maternal depression limit the generalizability of the findings. Accordingly, the results should be interpreted as evidence that proximal nurturing care resources were the strongest correlates of caregiver stimulation practices within this setting, rather than as evidence that socioeconomic determinants are unimportant, particularly within a country marked my nurturing care inequities like Brazil [15]. Despite these limitations, the study offers several practical implications for pediatric practice and primary healthcare. First, integrating book- and toy-lending or donation initiatives into primary healthcare may represent a feasible, low-cost strategy to promote caregiver engagement in developmentally stimulating activities.(16) Second, developmental guidance should become a more deliberate component of routine child health consultations, supported by training healthcare professionals in anticipatory guidance and developmental surveillance [16]. Finally, prenatal care represents an underused opportunity to introduce families to the principles of nurturing care before the child is born [17], reinforcing the continuity of developmental support across the life course [18].

Ultimately, the study provides practical evidence that promoting nurturing care need not depend exclusively on addressing socioeconomic determinants, but can also be advanced through evidence-informed, developmentally focused practices embedded within primary healthcare and pediatric services. Beyond its immediate clinical implications, the study makes an important conceptual contribution to the early childhood development literature. Whereas previous Brazilian studies have primarily examined predictors of early childhood developmental outcomes, Teixeira et al. shift attention to the caregiver behaviors that precede those outcomes. By integrating distal, intermediate, and proximal determinants within the Nurturing Care Framework, the study provides a useful model for investigating caregiver engagement in stimulation activities in diverse settings and offers a framework that can inform future implementation research.

## Data availability statement

NA.

## Conflicts of interest

The authors declare no conflicts of interest.
